# Aberrant histone modification and inflammatory cytokine production of peripheral CD4+ T cells in patients with oral lichen planus

**DOI:** 10.1111/jop.12790

**Published:** 2018-11-08

**Authors:** Jun Shen, Cao Yin, Xiao Jiang, Xuan Wang, Shujuan Yang, Guangbao Song

**Affiliations:** ^1^ Department of Oral Medicine Stomatological Hospital Southern Medical University Guangzhou Guangdong China; ^2^ Department of Oral Pathology Stomatological Hospital Southern Medical University Guangzhou Guangdong China

**Keywords:** acetylation, cytokine, histone modification, oral lichen planus, T cell

## Abstract

**Backgrounds:**

To investigate alterations in histone modification and histone deacetylases (HDACs) in patients with oral lichen planus (OLP), and to evaluate correlations with inflammatory cytokine production.

**Methods:**

Global histone H3/H4 acetylation and HDAC activity in CD4+ T cells from 23 patients with OLP and 10 healthy control subjects were examined using spectrophotometry. The mRNA levels of eight members of four classes of HDAC genes were measured by real‐time quantitative polymerase chain reaction. Forty cytokines involved in inflammation were examined with a cytokine array. The correlation between histone modification and cytokine production was analyzed.

**Results:**

Global histone H3 hypo‐acetylation was observed in OLP patients. Patients with OLP had significantly higher HDACs activity,and higher HDAC6 and HDAC7 mRNA level compared with the controls. Of the 40 cytokines in the cytokine array, eight were significantly increased in OLP patients: interleukin (IL)‐4, IL‐8, IL‐1ra, tumor necrosis factor receptor II (TNFR II), macrophage inflammatory protein 1b (MIP‐1b), fibrosis‐associated tissue inhibitors of metalloproteinase 1 (TIMP)‐1, monocyte chemotactic protein 1 (MCP‐1), and eotaxin‐2. In the OLP group, the acetylation level of histone H3 was negatively correlated with IL‐4 and MCP‐1 production, and the expression of HDAC6 mRNA was positively correlated with MCP‐1 production. In the non‐erosive subgroup, acetylation of histone H3 was negatively correlated with IL‐4, IL‐16, and TIMP‐2 production. In the erosive OLP subgroup, the expression of HDAC7 mRNA was positively correlated with MIP‐1a production.

**Conclusion:**

Aberrant histone modification of CD4+ T cells in peripheral blood could occur in OLP patients, and possibly affects inflammatory cytokine production.

## 1 INTRODUCTION

Oral lichen planus (OLP) is one of the most common chronic inflammatory disorders of the oral mucosa, characterized by T lymphocytes infiltration, chronicity, and female predilection.^1^, ^2^ Although the cause of OLP is not fully understood, there is considerable evidence suggesting that immune dysregulation is involved in the pathogenesis of OLP.^3^, ^4^ The immune dysregulation in OLP is reflected by the presence of CD4+ T cells and various inflammatory cytokines.^5^, ^6^ These cytokines are produced by a variety of cells under chronic inflammatory conditions, which in turn leads to the development of immune‐mediated inflammatory diseases. In OLP, several inflammatory cytokines, such as interleukin (IL)‐2, IL‐4, IL‐6, IL‐8, IL‐10, IL‐12, IL‐17, interferon (IFN)‐γ, and tumor necrosis factor (TNF)‐α, have been shown to have abnormal expression patterns.^7^, ^8^, ^9^ However, the production and regulation of cytokines is complex. As such, studies that have focused on single or several inflammatory cytokines have produced contradictory results, and the mechanisms of cytokine regulation and their role in the chronic inflammatory environment of OLP are not understood.

In recent years, epigenetic mechanisms have been shown to play fundamental roles in a variety of biological processes including cell growth, development, differentiation, and genomic stability. Epigenetic changes can result in gene dysregulation, leading to various pathological conditions such as cancer or autoimmune diseases.^10^, ^11^ Histone acetylation, a primary aspect of epigenetic mechanisms, plays key roles in remodeling chromatin conformation. The level of histone acetylation is highly dynamic and regulated by the opposing action of two enzyme families, histone deacetylases (HDACs) and histone acetyltransferases (HATs). HDACs remove acetyl groups from target histones, resulting in histone hypo‐acetylation that restores the positive charge of histones leading to a closed chromatin configuration, which may potentially induce further epigenetic changes and altered gene expression.^12^ There is increasing evidence that HDACs and their inhibitors may mediate the development of chronic inflammation by modulating the expression of many inflammatory cytokines and mediators such as IFN‐γ, TNF‐α, and interleukins.^13^, ^14^ These studies have highlighted the involvement of histone modification in the pathogenesis of various autoimmune diseases including rheumatoid arthritis (RA), systemic lupus erythematosus (SLE) and systemic sclerosis (SSc).^15^, ^16^, ^17^

To date, the involvement of epigenetic histone modification in the pathogenesis of OLP has not been examined. In this study, we investigated whether the histone modification pattern is altered in patients with OLP and examined the expression of eight members of four classes of HDACs in OLP CD4+ T cells. In addition, we also examined the relation of acetylation modification and peripheral blood cytokine levels in OLP.

## 2 MATERIALS AND METHODS

### 2.1 Patients

A total of 23 patients with OLP and 10 healthy volunteers were recruited from the Department of Oral Medicine at the Stomatological Hospital of Southern Medical University. All patients were clinically and pathologically diagnosed with OLP according to the World Health Organization (WHO) diagnostic criteria for OLP. Patients were subdivided into two groups: erosive type OLP (EOLP) and non‐erosive type OLP (NEOLP). The two OLP subgroups were matched for age and sex. In addition, the healthy individuals were also age and sex matched. Subjects with other oral or systematic disorders, those receiving immunotherapy, and those that received any medical treatment for OLP (local or systematic) within the 3 months prior to specimen collection were excluded from the study.

This study was approved by the Human Ethics Committee of the Southern Medical University, and written informed consent was obtained from all subjects. The whole experiment procedure was conducted in accordance with the Declaration of Helsinki. The clinical characteristics of the participants are shown in Table 1.

**Table 1:** Clinical features of the subjects

	n	Age (y)	Gender	
			Male	Female
OLP				
EOLP	12	43.7 ± 11.0	3 (25.0)	9 (75.0)
NEOLP	11	46.4 ± 13.2	3 (27.3)	8 (72.7)
Control	10	43.7 ± 12.1	2 (20.0)	8 (80.0)
Total	33	44.6 ± 11.8	8	25
Test statistics		*F *=* *0.181	*F *=* *0.157	
*P* value		*P *=* *0.836	*P *=* *0.925	

EOPL, erosive type oral lichen planus; NEOLP, non‐erosive type oral lichen planus; OLP, oral lichen planus.

### 2.2 Cell preparation

Peripheral blood mononuclear cells were isolated by Ficoll‐Hypaque density gradient centrifugation (Tianjin Haoyang Biological Manufacture Co., Ltd., Tianjin, China), and CD4+ T cells were isolated by negative selection using magnetic beads, according to the protocol of the manufacturer (R&D Systems, Inc., Minneapolis, MN). The purity of the CD4+ T cells was checked by flow cytometry and was typically >94%.

### 2.3 Histone extraction and detection of global histone H3/H4 acetylation

Isolation of histones was performed using a Total Histone Extraction Kit (Epigentek, Brooklyn, New York City, NY). Histone protein concentration was measured with the BCA™ Protein Assay Kit (Pierce, Rockford, IL). Global histone H3/H4 acetylation detection was performed using EpiQuik™ global histone H3/H4 acetylation assay kits according to the manufacturer's instructions (Epigentek). Briefly, acetylated histone H3/H4 was detected with a high‐affinity antibody, and the ratios and amounts of acetylated histone H3/H4 were quantified with a horseradish peroxidase‐conjugated secondary antibody color development system. Color was measured by absorbance at 450 nm.

### 2.4 Measurement of HDAC activity

Isolation of nucleoprotein was performed using the EpiQuik Nuclear Extraction Kit (Epigentek). Nucleoprotein concentration was measured with the BCA™ Protein Assay Kit (Pierce). HDAC activity was detected with the EpiQuik HDAC Activity Assay Kit (Epigentek).

### 2.5 RNA isolation, cDNA synthesis, and real‐time RT‐PCR

Total RNA was isolated from CD4+ T cells using the RNeasy Mini kit (Tiangen Biotech Co., Ltd., Beijing, China) according to the manufacturer's instructions. The RNA was stored at −80°C until use. First strand cDNA was synthesized using the Revert Aid™ first strand cDNA synthesis kit (Takara, Shiga, Japan). Real‐time semiquantitative reverse transcriptase‐polymerase chain reaction (RT‐PCR) was performed with Sybr Green master mix using an ABI Prism 7300 System. A series of five dilutions of 1 RNA sample were included to generate a standard curve, and this was used to obtain relative concentrations of the transcript of interest in each of the RNA samples. GAPDH was used as an endogenous control to normalize the amount of total RNA. Primers of eight members of HDACs genes were used (HDAC1, HDAC2, HDAC4, HDAC6, HDAC7, HDAC9, HDAC11, SIRT1), and that of GAPDH (Table 2).

**Table 2:** Primer sequences used in quantitative real‐time reverse‐transcriptase polymerase chain reaction (RT‐PCR) assays

Gene	Sense primer sequence(5′→3′)	Antisense primer sequence(5′→3′)
*GAPDH*	GATTCCACCCATGGCAAATT	TCTCGCTCCTGGAAGATGGT
*HDAC1*	ATCTATCGCCCTCACAAAGCC	TCCGACATGTTATCTGGACGG
*HDAC2*	ATATGGCTGTTAATTGGGCTGG	AATTCAAGGATGGCAAGCACA
*HDAC4*	CTCATCGTGGACTGGGACGT	GTCGTAGCGGTGGAGGGA
*HDAC6*	CTTCTGGTCCATCGGAGGATC	CTTCAATGCCCAGAGGGCTA
*HDAC7*	ACTTCCCTCTGCGCAAGACA	CCTTTCGGAGCAGTGGATTCT
*HDAC9*	GTCAGAAGTTCCTGTGGGCC	CTTCTCACGGACAACAGGGTC
*HDAC11*	GCTCGCCATCAAGTTTCTGTT	GAAGTCTCGCTCATGCCCAT
*SIRT1*	TTTCCATGGCGCTGAGGTAT	TCTGGCATGTCCCACTATCACT

### 2.6 Cytokine array

For cytokine array analysis, peripheral blood samples were collected from EOLP patients (n = 6) and NEOLP patients (n = 5), and age and sex matched to healthy controls (n = 5). The human cytokine antibody array (RayBiotech, Norcross, GA) capable of measuring 40 cytokines was used according to the manufacturer's instructions. Briefly, plasma was collected and stored at −80°C until use. While glass array slides were thawing to room temperature, the slides were blocked with 1× blocking buffer and incubated overnight at 4°C with 100 μL of plasma. Slides were washed and then incubated with 80 μL of biotin‐conjugated anticytokines at room temperature for 1‐2 hour. Slides were washed again and incubated at room temperature for 1 hour with Cy3 equivalent dye‐conjugated streptavidin. Each glass slide contained positive and negative internal controls. Fluorescence was detected with a GenePix 4000 scanner (RayBiotech, Sunnyvale, CA) using the Cy3 channel, and signal intensity was obtained with the scanning software. The RayBio Analysis Tool software (RayBiotech) was used to evaluate signal intensities after normalizing to the positive control and subtracting background noise. Changes in individual cytokines of ≥1.2‐fold increase and ≥0.8‐fold decrease were considered significant, as recommended by the manufacturer.

### 2.7 Statistical analysis

Experimental data were analyzed with the SPSS 24.0 software package and GraphPad Prism 7. Quantitative variables with a normal distribution were represented as in mean ± standard deviation ($\overset{¯}{x}+S$). A *t* test was used to compare means between two groups. With three or more groups, ANOVA was used to compare means and the LSD‐*t* test was used for multiple comparisons between means. Quantitative variables that were not normally distributed were represented as medians (*P*_25_~*P*_75_). Wilcoxon rank sum test and Kruskal‐Wallis *H* test were used to test the distribution of data, and the Spearman rank coefficient was used to determine the correlation between variables. The statistical significance was set at *P *≤* *0.05.

## 3 RESULTS

### 3.1 Aberrant global histone acetylation in OLP patients and controls

**Figure 1:**
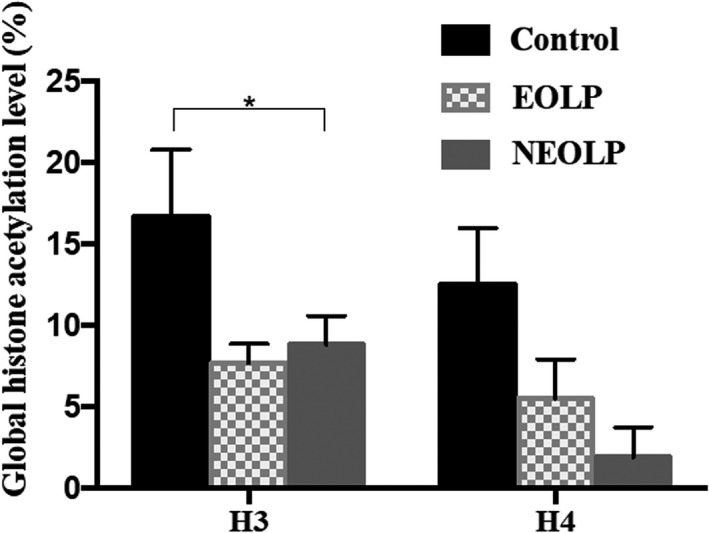
Comparison of the global histone acetylation level in peripheral blood CD4+ T lymphocytes between groups. *Statistically significant difference among the three groups. (Error bar: SEM)

Compared with the control group, the histone H3 acetylation level in the OLP group was significantly lower (*P *=* *0.0116). There was a significant difference in the histone H3 acetylation level among EOLP, NEOLP group and controls (*P *=* *0.025). No significant difference was found in histone H3 acetylation level between EOLP and NEOLP groups (*P *>* *0.05). No significant difference in the acetylation level of histone H4 was found between different groups (*P *>* *0.05) (Figure 1).

### 3.2 HDAC activity and related modifier HDAC genes

**Figure 2:**
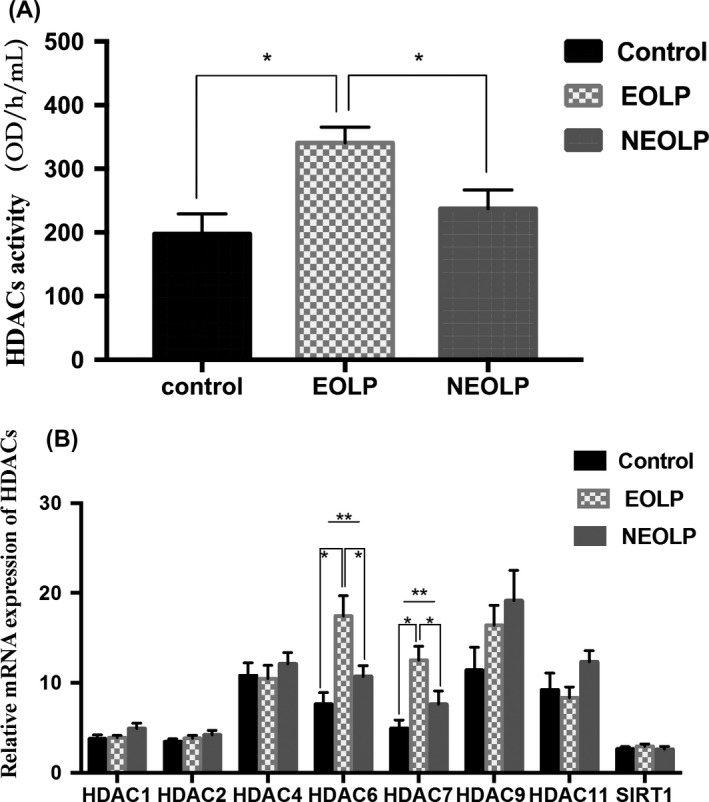
Comparison of HDAC activity (A) and relative mRNA expression of HDACs (B) in peripheral blood CD4+ T lymphocytes. *Statistically significant difference between 2 groups. ** Statistically significant difference between three groups. (Error bar: SEM)

Compared with the control, the HDAC activity in the OLP group was significantly higher *(P *=* *0.023). The HDAC activity in the EOLP was significantly higher than that in the NEOLP group and healthy control group (*P *=* *0.014 and *P *=* *0.001, respectively) (Figure 2).

The mRNA expression levels of related modifier HDAC genes were examined (Table 2). The mRNA expression of HDAC6 and HDAC7 was significantly increased in OLP patients as compared to controls (both, *P *=* *0.010). The mRNA expression of HDAC6 and HDAC7 in the EOLP group was higher that than in the NEOLP (*P *=* *0.009 and *P *=* *0.017, respectively) and the healthy control group (*P *<* *0.001 and *P *=* *0.001, respectively). Differences in the mRNA expression of other HDACs were not observed (all, *P *>* *0.05) (Figure 2).

### 3.3 Levels of inflammatory cytokines by cytokine array

**Figure 3:**
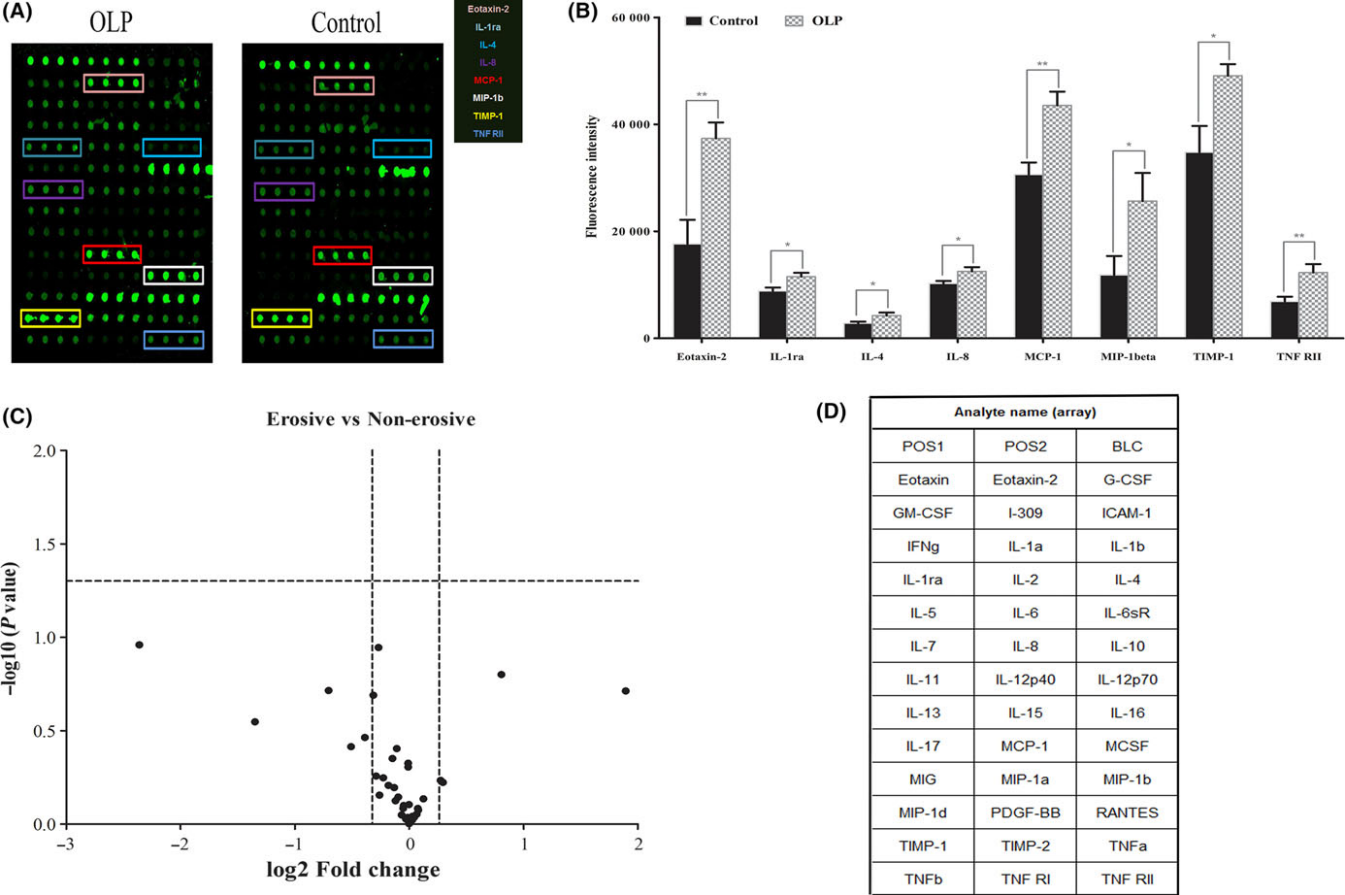
Antibody array‐based screening of oral lichen planus (OLP) sera for inflammatory cytokines. Serum samples from patients with OLP (n = 11) and healthy controls (n = 5) were applied to antibody‐coated slide arrays that can interrogate the levels of 40 proteins. A, Representative scanned fluorescence image of an antibody microarray. (Left): Microarray results of the OLP group; (Right): Microarray results of the control group. B, Of the proteins that were significantly increased in OLP sera, those that exhibited high fold‐change in the screening arrays are plotted as bar charts. *P <* *0.05, **P <* *0.01 (error bar: SEM). C, Volcano plot of the expression profiles of the 40 proteins expressed as a fold‐change (EOLP vs NEOLP), both expressed in log scales. D, The names and locations of each cytokine custom spots

Among the 40 cytokines in the array, eight were significantly increased in OLP patients compared with controls: IL‐4, IL‐8, IL‐1ra, TNFR II, MIP‐1b, TIMP‐1, MCP‐1, and eotaxin‐2. There were no significant differences in the cytokines examined between the EOLP and NEOLP groups (Figure 3).

### 3.4 Association of abnormal histone modifications with different cytokine levels

**Figure 4:**
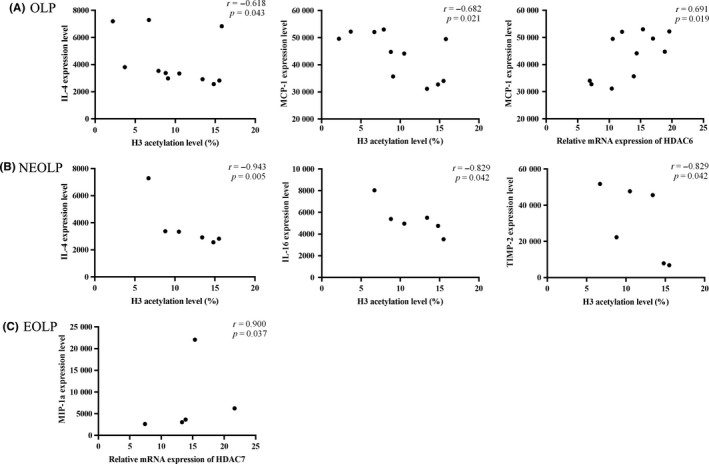
Associations of abnormal histone modifications with inflammatory cytokine production. A, In the OLP group, the acetylation level of histone H3 was negatively correlated with IL‐4 (a) (*r* = −0.618, *P *=* *0.043) and MCP‐1 (b) (*r* = −0.682, *P *=* *0.021) production, and the expression level of HDAC6 mRNA was positively correlated with MCP‐1‐production (c) (*r* = 0.691, *P *=* *0.019). (B) In the NEOLP subgroup, the acetylation level of histone H3 was negatively correlated with IL‐4 (d) (*r* = −0.943, *P *=* *0.005), IL‐16 (e) (*r* = −0.829, *P *=* *0.042), and TIMP‐2(f) (*r *= −0.829, *P *=* *0.042) production. (C) In the EOLP subgroup, the expression level of HDAC7 mRNA was positively correlated with MIP‐1a production (g) (*r* = 0.900, *P *=* *0.037)

As shown in Figure 4, the acetylation level of histone H3 in the OLP group was negatively correlated with IL‐4 (*r* = −0.618, *P *=* *0.043) and MCP‐1 (*r* = −0.682, *P *=* *0.021) production, and the expression level of HDAC6 mRNA was positively correlated with MCP‐1‐production (*r* = 0.691, *P *=* *0.019). In the NEOLP subgroup analysis, acetylation of histone H3 was negatively correlated with IL‐4 (*r* = −0.943, *P *=* *0.005), IL‐16 (*r* = −0.829,*P *=* *0.042), and TIMP‐2(*r* = −0.829,*P *=* *0.042) production. The expression level of HDAC7 mRNA was positively correlated with MIP‐1a production in the EOLP group (*r* = 0.900, *P *=* *0.037).

## 4 DISCUSSION

Histone modification and that of related modifying enzymes has received much attention because of their ability to affect the dynamic chromatin structure and regulate gene expression. It has been reported that histone modification is associated with several critical events in T cells, such as T cell development, activation, differentiation, and cytokine production.^18^, ^19^ In patients with OLP, large numbers of T lymphocytes accumulate beneath the epithelium of the oral mucosa, contributing to the differentiation of the stratified epithelium, hyperkeratosis, and erythematous lesions. In OLP local lesions, CD4+ T cells are the major lymphocytes present in the subepithelial and lamina propria and are clustered deeper down to form a typical lymphocyte‐rich band.^20^, ^21^ However, aberrant histone modification in T cells and the contribution to the pathogenesis of OLP remain unknown. In the present study, we showed for the first time that a histone of CD4+ T cells in peripheral blood was aberrantly modified and that the modification may be associated with the pathogenesis of OLP.

In this study, we found global histone H3 hypo‐acetylation and significant changes in the HDAC activity and expression of genes that regulate histone acetylation in CD4+ T cells of peripheral blood. The mRNA expression of HDAC6 and HDAC7 was significantly increased in OLP CD4+ T cells. Since HDACs remove acetyl groups from target histones, up‐regulation of HDAC7 and HDAC6 expression causes increased deacetylation, and subsequent aberrant hypo‐acetylation. Previous studies have demonstrated aberrant histone modification is involved in autoimmune diseases and inflammatory disorders.^22^, ^23^ In OLP research, a recent study provides evidence that acetylation modification of histone H3 at lys9 in lesion tissues is associated with poor response to therapy.^24^ Findings of these prior studies, in combination with the results of the current study, suggest that aberrant histone modification may play an important role in the pathogenesis of many autoimmune disorders, including OLP. Abnormal global H4 acetylation in CD4+ T was not observed in this study, suggesting that dysregulation of global histone acetylation may not be a general feature in CD4+ T cells.

Aberrant histone modification by HDACs may modulate the inflammatory response by regulating many cytokines and mediators. To this end, we examined which cytokines may be modulated by histone modification in CD4+ T cells in peripheral blood of OLP patients. Although there have been a large number of publications in recent years identifying specific abnormal cytokines in OLP, it is becoming increasingly clear that cytokines do not work separately, but rather interact and function in complex immunomodulation networks. We used a high‐throughput array system to detect 40 cytokines in the OLP microenvironment, and a notable increase was observed in eight proteins. Importantly, five of the eight cytokines (eotaxin‐2, IL‐1ra, MCP‐1, MIP‐1b, and TNFRII) have not been reported in OLP before. Compared with previous studies, however, we did not find Th1/Th2 or other Th subsets predominant in OLP. The different results may be due to the different methods of detection, or the influence of differences in genetic background, lesion site, sex, and age between study populations.

We eventually identified five cytokines, IL‐4, IL‐16, MCP‐1, TIMP‐2, and MIP‐1a that may be associated with histone modification. The level of HDAC6 mRNA expression positively correlated with MCP‐1 production in the OLP group and the level of HDAC7 mRNA expression positively correlated with MIP‐1a production in the EOLP group. HDAC6 and HDAC7 are both class II HDACs that have been found to be correlated with pathologic grade and tumor stage in several human cancers.^25^, ^26^ They also appear to have crucial roles in T cell development and function. HDAC6 overexpression results in increased T cell migration and chemotaxis,^27^ and nuclear export of HDAC7 regulates the expression of cytokines, cytokine receptors, and adhesion molecules.^28^ A selective class II HDAC inhibitor has been found to decrease inflammatory cytokine levels in different cell lines.^29^, ^30^ We can infer that the inhibition of HDAC activation, and the subsequent modification of chromatin status, change the molecular signature of mediators, leading to changes in the inflammatory environment.

In summary, our results revealed that histone modifications are apparently altered in peripheral blood of CD4+ T cells and are correlated with cytokine production in OLP patients. These findings provide novel insights into the pathogenesis of OLP. It is possible that the reversal of epigenetic changes could be an effective therapy for OLP. Further research is necessary to confirm our results, and to determine the causes leading to the deregulation of epigenetic mechanisms in patients with OLP.

## CONFLICT OF INTEREST

The authors declared that they have no conflict of interest.
